# Informing Implementation of a National Integrated Clinical Pathway for Low Back Pain in Ireland: A Pre‐Implementation Qualitative Study With General Practitioners

**DOI:** 10.1002/msc.70030

**Published:** 2025-01-03

**Authors:** Cathriona Murphy, Helen French, Geraldine McCarthy, Bianca Albers, Caitriona Cunningham

**Affiliations:** ^1^ Physiotherapy Department University Hospital Kerry Tralee Ireland; ^2^ School of Physiotherapy Royal College of Surgeons in Ireland Dublin Ireland; ^3^ School of Medicine University College Dublin Dublin Ireland; ^4^ Department of Rheumatology Mater Misericordiae University Hospital Dublin Ireland; ^5^ Institute for Implementation Science in Health Care University of Zurich Zurich Switzerland; ^6^ School of Public Health Physiotherapy and Sports Science University College Dublin Dublin Ireland

**Keywords:** barriers and facilitators, clinical pathways, general practitioners, implementation science, low back pain, qualitative research

## Abstract

**Introduction:**

Ireland's Health Service Executive is developing a new national integrated low back pain (LBP) pathway spanning primary and secondary care to improve LBP healthcare. Clinical pathways are frequently employed to optimise clinical outcomes and resource use but are challenging to implement. Context‐specific implementation planning, leveraging implementation science and its conceptual frameworks, should inform successful implementation.

**Aim:**

To explore General Practitioner (GP) perceptions of the new LBP pathway, using their on‐the‐ground knowledge to assess barriers and facilitators to implementation with the goal of developing tailored implementation strategies.

**Methods:**

Qualitative data will be collected through semi‐structured one‐to‐one interviews with a purposive sample of GPs. Data analysis will be guided by Framework Analysis with initial open coding followed by mapping to the domains and constructs of the Consolidated Framework for Implementation Science and Proctor's implementation outcomes of acceptability, appropriateness and feasibility. Potential implementation strategies will be devised from the identified determinants of implementation.

**Conclusion:**

System‐wide changes and improvements in healthcare are difficult to accomplish. The rationale and design of a pre‐implementation study with key stakeholders is outlined with the view of informing optimal implementation of Ireland's new national integrated LBP pathway in primary care settings.

## 1 Introduction

Low back pain (LBP), the most prevalent musculoskeletal (MSK) disorder, remains the leading cause of years of disability worldwide and carries a high clinical and economic burden (Fatoye et al. 2023; Ferreira et al. 2023; Hurwitz et al. 2018; Wu et al. 2020). The persistent evidence‐practice gap in LBP care is indicative of healthcare systems struggling to deliver high‐quality care, a challenge compounded by healthcare cost inflation, workforce shortages, and rising demand from a growing and ageing global population (Foster et al. 2018, Liu et al. 2022; OECD 2023). In the face of these challenges, re‐evaluation of care delivery to minimise low‐value care has contributed to the increasing use of clinical pathways (CPs) to facilitate evidence‐informed practice, maximise patient outcomes and optimise resource use and cost‐effectiveness (Rotter et al. 2019). Low value care is of limited or no value to patients, thereby wasteful of limited resources, and includes unnecessary diagnostic testing and imaging, ineffective treatments and inefficient health system organisation (Hartvigsen, Kamper and French 2022; Zadro and Maher 2022). Clinical pathways, also known as critical pathways, care pathways, or care maps, are structured care plans describing essential steps in multidisciplinary care provision for patients with a specific condition (Rotter et al. 2010). Clinical pathways for LBP are associated with improved service efficiency, reduced wait times for patients and high levels of patient satisfaction (Murphy et al. 2022).

In Ireland, National Clinical Programmes have been developed by the Health Service Executive (HSE), the organisation responsible for public health service provision, to lead the development of health improvement initiatives to enhance care quality and access (Shaw 2020). Central to the quality improvement work of the National Clinical Programmes is the development of ‘modernised care pathways’ aiming to improve access to scheduled care services and reduce waiting times for 72 specific conditions, including LBP (HSE n.d.). The National Integrated Low Back Pain Pathway (NILBPP) was developed collaboratively by the National Clinical Programme for Trauma and Orthopaedic Surgery (NCPTOS) and the National Clinical Programme for Rheumatology (NCPR).

Until now, there have been no national clinical LBP guidelines in Ireland. Under usual care, general practitioners (GPs) are ordinarily the gatekeepers to elective Irish public healthcare services for the person with LBP, with onward referral options including primary care physiotherapy and secondary care consultant‐led orthopaedic and rheumatology services. Since 2011, physiotherapy‐led musculoskeletal (MSK) triage services have been used to address long outpatient waiting lists for orthopaedic and rheumatology consultant services, but direct referral by GPs to MSK triage services is not available. Through the triage services, clinical specialist physiotherapists assess patients on the orthopaedic/rheumatology waiting lists in hospital‐based, and more recently community‐based, triage clinics under the clinical governance of orthopaedic and rheumatology consultants. These triage services discharge > 80% of patients at their first appointment, suggesting that, contrary to LBP best practice guidelines, community‐based management of MSK conditions is not being optimised prior to secondary care referral (Fennelly et al. 2018). This echoes earlier research that reported frequent onward referral at first GP consultation with acute LBP in Ireland, as well as high use of radiographic imaging, inconsistent with European clinical guidelines (Fullen et al. 2007).

The modernised care pathways reflect the HSE's objective to optimise community‐based care and alleviate pressure on hospital services (Burke et al. 2018). GPs are the first point of contact in the new pathway with guidance to assist appropriate onward referral to primary or secondary care services, with onward referral via a new spine speciality specific e‐referral form. Clinical decision‐making tools such as STarT Back (Hill et al. 2010) and Spondyloarthritis Diagnosis Evaluation (SPADE) tool (www.spadetool.co.uk) are recommended in the pathway to assist GP decision‐making regarding onward referral. The pathway includes direct referral to MSK triage services for both GPs and primary care (PC) physiotherapists. The MSK triage clinical specialist physiotherapists screen e‐referrals to their service to determine if the person requires an in‐person assessment with them in their ‘triage and treat’ clinic or if they can be directed to primary care physiotherapy services in the first instance. The MSK triage clinical specialist physiotherapists access specialist spine consultant opinion as required with case discussion via in‐person or virtual interface clinics, the latter being particularly innovative and beneficial for hospital sites that do not have on‐site spine surgical services. Following the interface clinic, hospital consultant in‐person appointments can be scheduled as necessary with relevant specialities, including orthopaedic spine surgery, rheumatology, neurology and pain medicine. An MRI scan must be completed prior to an in‐person spine surgeon appointment. The new pathway should optimise both primary care management and use of consultant clinics, whilst simplifying the journey for patients requiring specialist opinion.

The NILBPP begins when a person with LBP consults their GP. As gatekeepers for the NILBPP, GP engagement with the pathway is critical for successful implementation and forms the focus of this proposed study. Clinical pathways as complex interventions pose a recognised implementation challenge (Pfadenhauer et al. 2017; Vanhaecht et al. 2012), compounded in this instance by evolving changes in primary care and GP resource pressures. The modernisation agenda in Irish healthcare with enhanced primary care provision for more complex and chronic conditions has increased GP workloads (Burke et al. 2018; Keenan et al. 2023; McDonnell et al. 2021). Ireland has one of the lowest ratios of GPs per 10,000 population in Europe (Teljeur et al. 2014) and high levels of emotional exhaustion have been reported amongst overworked GPs in Ireland (O'Dea et al. 2017). Ireland's lack of universal coverage of primary healthcare is unique amongst European countries. GPs in Ireland are independent contractors, and the majority of the population pay out of pocket for their services; GPs are reimbursed by the HSE for care provided to the remaining 43% of the population entitled to free GP care (Prior, Duff, and Scott 2019). This private contractor status confers greater independence and autonomy, and recent research into National Clinical Programme implementation in Ireland highlighted concerns that GPs may have limited capacity to fully engage with new care pathways without additional funding (Darker et al. 2018). GP practices, as independent, private businesses, may also vary in organisational structures, cultures and working practices. This highlights the need for pathway‐specific pre‐implementation engagement with GPs to explore the concordance between the new national pathway and their capacity to implement it. This study’s methods will be underpinned by implementation science to identify determinants of implementation from the perspective of GPs to support the development of tailored implementation strategies to promote effective pathway adoption. Such pre‐implementation preparation and planning was identified as an important contributor to successful implementation by a systematic review of the evidence‐to‐practice gap in primary care (Lau et al. 2016). Pre‐implementation consultation with stakeholders allows them to contribute to implementation planning, thereby enhancing engagement with the new intervention and building implementation capacity (Goodrich et al. 2020).

## 2 Implementation Framework and Outcomes

A thorough barrier and enabler analysis will facilitate a detailed understanding of the implementation context to inform tailored implementation strategies. The Consolidated Framework of Implementation Research (CFIR) will be used to assist in the identification and categorisation of contextual factors likely to influence the successful implementation of the NILBPP in Irish general practices. The CFIR, a determinant framework, was chosen for its comprehensiveness, suitability to qualitative research, and utility in mapping identified determinants of implementation to defined implementation strategies when combined with the Expert Recommendations for Implementing Change (ERIC). It focuses on identifying potential barriers and facilitators to implementation success across five contextual domains comprising 37 implementation constructs, drawing on knowledge from previous research across multiple fields including innovation, organisational change, implementation and knowledge translation (Damschroder et al. 2009). Its use in qualitative research is enabled by the provision of a qualitative codebook and guidance on CFIR use in conducting qualitative data collection and analysis (CFIR n.d.). Furthermore, the CFIR has demonstrated utility in the evaluation of barriers and facilitators of complex interventions in primary care (Harry et al. 2019; Pestka et al. 2022; Rogers et al. 2021; Roseen et al. 2024; Smith et al. 2022).

This study will additionally use three of Proctor's implementation outcomes commonly used in early‐stage implementation evaluations: acceptability, appropriateness and feasibility (Proctor et al. 2011, 2023). These are perceptual outcomes, therefore appropriate for this qualitative evaluation (Lyon and Bruns 2019). The outcomes are defined as follows: (1) *acceptability:* perception among implementation stakeholders that a given treatment, service, practice or innovation is agreeable, palatable, or satisfactory; (2) *appropriateness:* perceived fit, relevance or compatibility of the innovation for a given practice setting, provider or consumer; and/or perceived fit of the innovation to address a particular problem or issue; (3) *feasibility:* extent to which a new treatment or innovation can be successfully used or carried out within a given study (Proctor et al. 2011).

## 3 Aim and Objectives

This research forms part of a wider project to inform LBP service improvement in Ireland, including a systematic review of published LBP clinical pathways and a survey of key stakeholders (Murphy et al. 2022, 2024a, 2024b). This study aims to elicit GPs' perspectives on the Irish National Integrated LBP Pathway to inform and optimise its implementation in primary care. The specific objectives include,

1. To explore GPs' perceptions of the acceptability, appropriateness and feasibility of the national integrated LBP pathway;
2. To identify potential enablers and barriers to pathway implementation in primary care;
3. To map the identified enablers and facilitators in a comprehensive and systematic manner to the domains and constructs of the Consolidated Framework for Implementation Research (CFIR);
4. To systematically match the identified implementation determinants with suitable implementation strategies.

## 4 Study Design and Theoretical Orientation

A qualitative study design will be used, with data collection via semi‐structured interviews with individual GPs. The qualitative study design and analysis are informed by critical realist ontology and the epistemological framework of contextualism. This reflects the researchers' understanding that the knowledge generated from this work is contextual with an interpretative element, but nevertheless reflects an underlying ‘reality’ (Braun and Clarke 2013).

## 5 Participants and Recruitment

A purposive sample of GPs will be recruited to ensure both urban and rural representation. GPs will be invited to join the study via email leveraging researcher networks, which include established GP contacts of the research team members, the University College Dublin (UCD) GP Network, the National Clinical Programme for Trauma and Orthopaedic Surgery, the National Clinical Programme for Rheumatology and the National Clinical Lead for Back and Neck Pain Referral Pathways. The email invitation will include a participant information sheet with details regarding: the purpose of the research, what participation will entail, the approximate time commitment required, data protection and the research team. Interested GPs will contact the research team directly and will then be provided with a consent form via email, with both postal and electronic options for signing.

The estimated sample size for this study, 10–15 GPs, was determined with consideration of the concept of ‘information power,’ which uses five different study characteristics to assess whether larger or smaller sample sizes are required to ensure sufficient information power: study aim, sample specificity, established theory, quality of dialogue and analysis strategy (Malterud, Siersma, and Guassora 2016). The proposed sample size is considered adequate given the unidisciplinary nature of the study (sample specificity), the well‐defined subject matter (study aim) and the use of an implementation framework to systematically catalogue the findings (use of established theory). It is also consistent with recently published musculoskeletal‐related qualitative research with GPs (Larsen et al. 2024; Okwera and May 2019; Slatman et al. 2022). The sample size will be revisited during the research process, particularly with respect to the quality of the information garnered from the participant interviews (quality of dialogue) to ensure final sample adequacy (Malterud, Siersma, and Guassora 2016).

## 6 Data Collection

Qualitative data will be collected through semi‐structured, audio‐recorded, one‐to‐one interviews with participating GPs. The option of a telephone interview will be available depending on each participant's preference. Interviews were chosen to facilitate an in‐depth exploration of each participant's opinions of the new pathway. A specific interview topic guide has been developed by the research team with reference to the CFIR, Proctor's taxonomy of implementation outcomes and the findings of a prior survey from this research team eliciting the views of clinical specialist physiotherapists on LBP care in Ireland (Murphy et al. 2024a, 2024b). The topic guide will aid consistency of approach but may be iteratively refined during the data collection period to facilitate exploration of any new discussion points that might arise. Relevant demographic characteristics, including participants' years of experience, location and size of practice, and prior training in MSK medicine, will be documented at the interview outset.

The research team comprises a practicing clinical specialist physiotherapist/PhD candidate (CM), two physiotherapy academics with extensive research and musculoskeletal clinical experience (CC and HPF), and a consultant rheumatologist/clinical professor of medicine (GM). The principal investigator (PI), CM, with training in qualitative research and implementation science, will conduct the interviews. Each interview will be conducted via Zoom online teleconferencing software (or telephone) on a day and time convenient to the participant. Participants will be informed that they can stop the interview and withdraw from the study at any time. Each interview, with the participant's agreement, will be audio‐recorded. At the end of each interview, the researcher will document their reflections in field notes. Data from each interview will be transcribed verbatim and interviewees will have the option to review a transcript of their interview with the opportunity to edit their answers prior to the commencement of data analysis.

## 7 Data Analysis

The 7‐stage framework analysis outlined by Gale et al. (2013) will guide the qualitative data analysis. As outlined in Table 1, analysis will involve initial open inductive coding followed by mapping to the CFIR domains and constructs, as well as Proctor's implementation outcomes, facilitating both inductive and deductive coding/theme development. Inductive coding ensures a comprehensive and thorough approach that allows for the identification of themes and determinants of implementation that may be overlooked in a solely deductive analysis. Incorporating the CFIR facilitates categorisation of implementation determinants in a systematic manner, drawing on previous research across multiple fields, and contributes to the growing IS knowledge base. NVivo (QSR International Pty Ltd.), qualitative data analysis software, will be used to assist data management. A list of potential implementation strategies will be devised by matching the identified CFIR determinants of implementation to implementation strategies using the CFIR‐ERIC matching tool (Waltz et al. 2019). This preliminary list may require further evaluation and tailoring through expert/stakeholder review to develop priority recommendations for inclusion in an implementation plan.

**TABLE 1:** Seven stage framework analysis to be used in this study (informed by Gale et al. 2013).

1	Transcription	Each interview will be transcribed verbatim and transcripts cleaned of potentially identifiable information.
2	Familiarisation with the interview	Researchers will re‐read the transcripts and associated field notes for familiarisation (CM and CC).
3	Coding	Three transcripts will be openly, independently coded line by line by two research team members (CM and CC).
4	Developing a working analytical framework	The resultant codes will be reviewed by all research team members to develop a code set and analytic framework.
5	Applying the analytical framework	The analytic framework will be applied in the analysis of the remaining transcripts through indexing transcripts using the existing codes. This will be an iterative process with additional codes developed if required (CM). Any additional codes will be discussed and agreed with another team member (CC).
6	Charting data into the framework	Data will be charted into the framework matrix, allowing for summation of the data by category, with the identification of illustrative quotes (CM).
7	Interpreting the data	The data will be interpreted to develop subthemes/themes (CM and CC). These will be refined by whole group discussion and then mapped, where applicable, to the CFIR constructs and domains to identify multilevel barriers and enablers to implementation, and to Proctor's implementation outcomes of acceptability, appropriateness and feasibility.

Abbreviation: CFIR, Consolidated Framework of Implementation Research.

## 8 Reflexivity

The PI and research team members are independent researchers and are not involved in the implementation of the national pathway. The PI will actively engage in reflective practice throughout the research process to minimise potential sources of bias, including their previous participation in multi‐professional focus groups to inform the development of the new national pathway. Reflective notes will be made at the conclusion of each participant interview. The use of independent coding and collaborative development and refinement of themes amongst the research team will also minimise potential bias.

## 9 Quality

To enhance quality and trustworthiness, this research protocol has been developed with reference to Tracy's ‘big tent’ criteria of qualitative methodological quality (Tracy 2010). These criteria are comprehensive but flexible, as they can be applied across different qualitative paradigms (Tracy 2010). In reference to the eight ‘big tent’ criteria, the subject matter is relevant and timely with national significance (worthy topic). Rich rigour is demonstrated through explicitly outlining theoretical constructs, as well as data collection and analysis procedures. Additionally, both field notes and transcripts will be used in data analysis and an audit trail will be kept through NVivo (QSR International Pty Ltd.) data analysis software for transparency in decision‐making. Sincerity will be enhanced by ensuring that the transcripts accurately reflect the interview audio recordings, the keeping of field notes, and engaging in self‐reflexive practice during all stages of the research process. The use of independent coding by two research team members and the collaborative development and refinement of themes enhances credibility, as will the use of illustrative supporting quotes from multiple participants in the presentation of findings. Ethical considerations are outlined below. In preparation of a research paper for submission to a peer‐reviewed journal, the write‐up will be undertaken with consideration of the remaining three ‘big tent’ criteria: resonance, significant contribution and meaningful coherence. In addition, the research findings will be reported consistent with the Standards for the Reporting of Qualitative Research (O'Brien et al. 2014).

## 10 Ethical Considerations

### 10.1 Consent to Participate

GPs invited to participate will be emailed a participant information sheet specifying the aims and objectives of the study, what participation will involve, confidentiality measures in data management and dissemination, that they can withdraw from the study at any time and providing the opportunity to ask further questions regarding the study and their potential participation. Prior to enrolment in the study, each volunteer will sign an informed consent form, which includes the request to record their interview. At the beginning of each interview, the PI will inform participants of the study aims and objectives and remind them of their right to terminate the interview and to opt‐out of the study at any time. Participants, all private practitioners, will be offered a small honorarium to compensate for their time.

### 10.2 Data Management

Data will be processed and stored in accordance with UCD's Research Data Management Policy and General Data Protection Regulation (GDPR) requirements. The audio files and original transcripts will be stored securely in Google Drive, a cloud‐based storage service approved by UCD for research data storing and sharing purposes. Only the PI will have access to these files, and they will be deleted on study completion. Each participant will be assigned a study ID for pseudonymisation, and their interview transcript will be cleaned of any identifying information by the PI before the de‐identified transcripts are shared via Google Drive with members of the research team for data analysis purposes. Quotes from interviews may be used in publications or presentations arising from this study, but no identifying information will be featured.

### 10.3 Ethical Approval

This qualitative study is considered a minimal risk study due to its minimally invasive nature and all participants being qualified healthcare professionals. Ethics approval has been granted by the UCD Human Research Ethics Committee (LS‐C‐24–349).

## 11 Dissemination

This study will be written up and a paper submitted for consideration for publication in an international peer‐reviewed journal and for presentation at national conferences and health service events relevant to key LBP pathway stakeholders, including people with MSK pain, GPs, physiotherapists, consultant spine surgeons and rheumatologists. It will also be presented at pertinent international conferences. Furthermore, the findings and those from companion studies completed by the research team (Murphy et al. 2022, 2024a, 2024b) will be compiled to develop recommendations for the improvement of integrated LBP healthcare services in Ireland, which will be shared with the NCPTOS and the NCPR.

## 12 Discussion

System‐wide changes and improvements in healthcare are difficult to accomplish. Successful nationwide uptake of the new NILBPP will require careful preparation and targeted, context‐specific implementation planning informed by key stakeholder engagement. Implementation science and its conceptual frameworks can be harnessed to optimise this process, providing an evidence‐based, comprehensive and systematic approach. We have outlined the rationale and design of a pre‐implementation study with GPs, exploring their perceptions of the new pathway and their capacity to enact it, using their on‐the‐ground knowledge to assess barriers and facilitators to implementation with the goal of developing tailored implementation strategies.

## Author Contributions

**Cathriona Murphy**: conceptualisation, methodology, writing‐original draft, writing‐review and editing. **Helen French**: conceptualisation, methodology, supervision, writing‐review and editing. **Geraldine McCarthy**: funding acquisition, conceptualisation, methodology, supervision. **Bianca Albers**: methodology, writing‐review and editing. **Caitriona Cunningham**: conceptualisation, methodology, supervision, writing‐review and editing.

## Conflicts of Interest

The authors declare no conflicts of interest.

## Data Availability

Data sharing is not applicable to this article as no new data were created or analysed in this study.
